# Prenatally Diagnosed Infantile Myofibroma of Sartorius Muscle—A Differential for Soft Tissue Masses in Early Infancy

**DOI:** 10.3390/diagnostics11122389

**Published:** 2021-12-18

**Authors:** Ștefan Popa, Dan Apostol, Ovidiu Bîcă, Diana Benchia, Ioan Sârbu, Carmen Iulia Ciongradi

**Affiliations:** 1 3rd Department of Medical Specialities–Legal Medicine, “Grigore T. Popa” University of Medicine and Pharmacy Iași, 700115 Iași, Romania; dr_popastefan@yahoo.com; 2 “Sfânta Maria” Emergency Children Hospital Iași, 700309 Iași, Romania; dan12apostol@yahoo.com (D.A.); ovidiubica.d@gmail.com (O.B.); diana_benchia@yahoo.com (D.B.); iuliaciongradi@yahoo.com (C.I.C.); 3 2nd Department of Morphofunctional Sciences–Cell and Molecular Biology, “Grigore T. Popa” University of Medicine and Pharmacy Iași, 700115 Iași, Romania; 4 2nd Department of Surgery–Pediatric Surgery and Orthopedics, “Grigore T. Popa” University of Medicine and Pharmacy Iași, 700115 Iași, Romania

**Keywords:** infantile myofibromatosis, lower limb tumor, thigh tumor, prenatal diagnose

## Abstract

Background: Infantile myofibromatosis (IM) is a soft tissue disease with solitary or multiple benign tumors, and an etiology still unknown. IM is a mesenchymal disorder of early infancy and is more frequent in males. IM may present as a solitary lesion of the skin, bone, muscle, subcutaneous tissue, located at the head, neck, and trunk, with good prognosis; or, as a multicentric form, with or without visceral involvement (heart, lung, gastrointestinal tract, kidney), with a poor prognosis. The definitive diagnosis of IM is confirmed by pathology. Treatment may be conservative, surgical, or chemotherapeutical. Case presentation: A two months old female patient, prenatally diagnosed at 30 weeks, presenting with a tumor on the antero-internal aspect of the left thigh. She was admitted due to rapid postnatal evolution, and the patient required surgery for tumor resection. Previously, clinically, biological and imaging investigations were performed, but the final diagnosis was histological and by immunostaining. The patient had a favorable postoperative outcome. Conclusions: Despite its low frequency, IM should be considered in the differential diagnosis of soft tissue masses at an early age. The clinical form (solitary or multicentric), location, and visceral involvement will dictate the treatment and prognosis.

## 1. Introduction

Infantile myofibromatosis (IM) manifests as a single or multicentric lesion of benign nature [1]. The incidence of the disease is low, although it is considered the most common type of mesenchymal tumor during infancy and early childhood, with an unknown etiology [2]. Sporadic presentation is more common in children < 2 years.

This article describes a case of a 2 months old girl who was detected with a left thigh mass on the prenatal ultrasound at 30 weeks of gestation; she was presented for consultation due to the presence and rapid growth of the tumor. The aim of this article is to present the clinical case, its management and to review the literature on this disease.

## 2. Case Report

**Figure 1:**
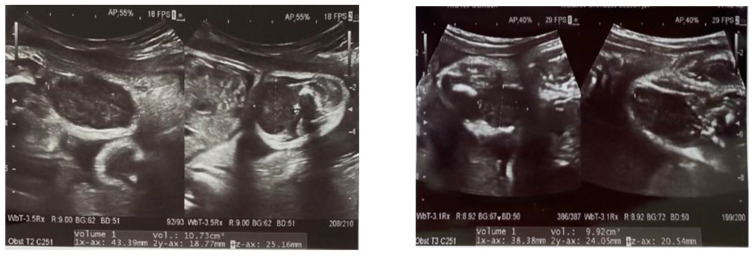
Prenatal ultrasound images at 30 weeks of gestation, showing an oval mass, hypoechoic with calcification on the thigh, with no evidence of bone involvement.

We report the case of a 2 months old female, presented for consultation due to the presence of a lump on her left thigh, with progressive and constant growth after birth. The lesion was first described on the prenatal ultrasound at 30 weeks of gestation as a pre-femoral soft tissue mass of 20/7 mm (Figure 1). The patient was delivered by cesarean section due to fetal distress but was otherwise normal at birth. Development was normal, and there was no relevant family history. On clinical examination, there was a 25/10 mm nodule on the antero-intern side of the left thigh that was firm, mobile and within the deep layers. The overlying skin was normal. There were no other lesions elsewhere on the patient’s body.

**Figure 2:**
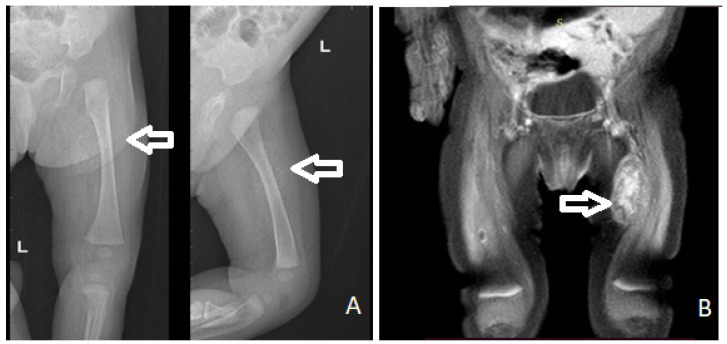
X-ray (**A**) and MRI (**B**) showing a mass on the antero-intern part of the left thigh (arrow).

The initial X-ray and ultrasound (US) showed a pre-femoral soft tissue mass that measured approximately 30/13 mm, with nonhomogeneous structure, hypoechoic areas, calcifications, and weak Doppler signal, being located anteriorly to the vascular elements of the thigh (Figure 2A). Abdominal ultrasound was normal.

Magnetic resonance imaging (MRI) showed a mass of 19.33/15.19/34 mm, with a nonspecific vascular involvement (Figure 2B). In T1-weighted images, the MRI appearance consisted of a low signal. In T2-weighted fat-saturated images, a high signal intensity of the lesion was shown with nonhomogeneous contrast setting after intravascular contrast was administered, but with late homogenization, located on the antero-internal part of the left thigh with an important mass effect on the left vastus intermedius muscle. The lesion was considered to be probably a schwanoma of the left saphenous nerve.

**Figure 3:**
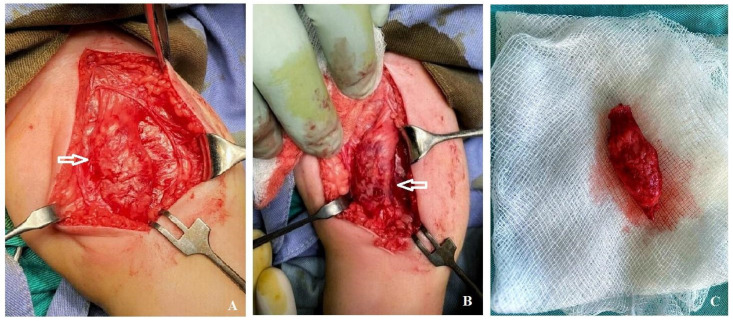
(**A**) Intraoperative aspect of the tumor. Note that the lesion includes the entire thickness of the sartorius muscle (arrow). (**B**) Tumor in the crural fascia that highlights an approximately 4 cm × 1.5 cm × 1.5 cm lesion which includes the entire thickness of the sartorius muscle (arrow). (**C**) Sartorius muscle resected at a distance of about 2 cm distal and proximal to the tumor, with complete tumor resection.

Elective surgery was scheduled. An italic S-shaped incision on the antero-internal face of the left thigh was performed, from the crural arch distally extended for about 6 cm. A mass of approximately 4 cm × 1.5 cm × 1.5 cm was revealed, which included the entire thickness of the sartorius muscle (Figure 3A,B). In the 1/3 medial part of the tumor, dissection was performed, isolating it from the femoral vasculo-nervous package without opening the sheath of the vasculo-nervous canal. The sartorius muscle was resected at a distance of about 2 cm distal and proximal to the tumor, with complete tumor resection (Figure 3C). Hemostasis was performed and adjacent tissue approximated. The excised mass was sent for pathological analysis. The patient had a favorable surgical outcome and was discharged 3 days postoperatively. At one year after surgery follow up, the child had no recurrence.

**Figure 4:**
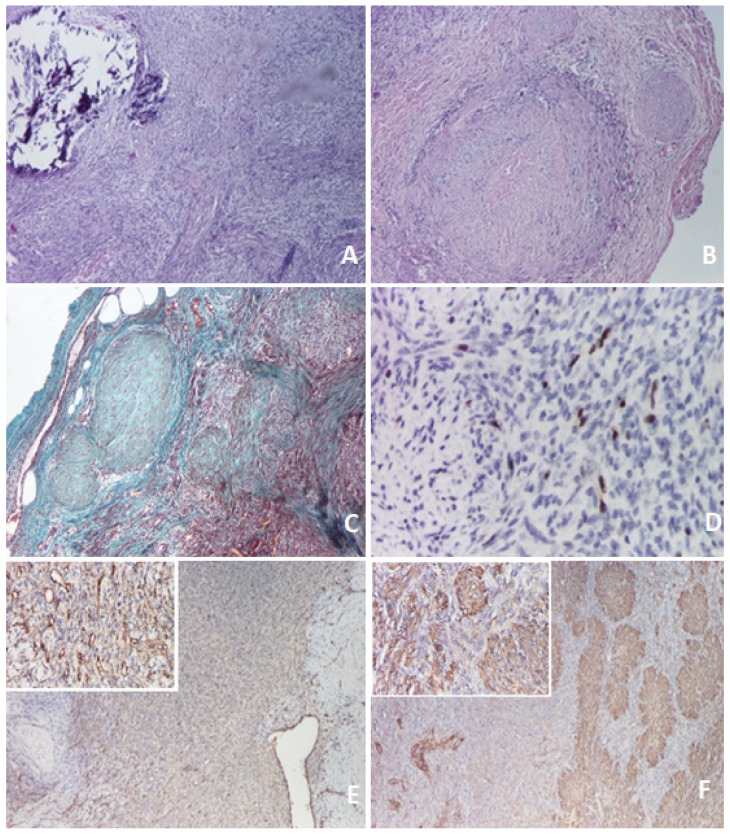
Hemathoxilin eosine staining (×40): (**A**) Multiple fusiform cells, arranged in fascicle and nodules. (**B**) Node with central necrosis. Trichromic Szekely stain (×40): (**C**) Biphasic aspect. Immunochemistry: (**D**) Ki67 + in about 5% of the tumor (×200). (**E**) CD34—vascular hemangiopericytoma-like proliferation (×40/×200). (**F**) Smooth muscle actin stain miotic nodule (×40/×200).

Histologically, the mass in the sartorius muscle was noted as a proliferation of tapered cells arranged in an irregular spiral pattern and crossed by thin-walled vessels. In the central region, biphasic proliferation consisting of nodules with necrosis and central calcifications was observed, and between the nodules, fusiform cell proliferates arranged in small intersecting bundles (Figure 4A,B). Occasional micronuclei were evident. A pseudocapsule formed by a thin layer of connective tissue <1 mm covered the mass. Neoplastic proliferation encompassed residual skeletal muscle fibers in the center of the lesion. Extracapsular and peripheral scarce mature adipose tissue were seen with isolated large-caliber blood vessels. Immunostaining revealed the following results: vimentin positive, smooth muscle actin positive in nodules with necrosis and calcifications (miotic nodules), desmin focal positive, Ki67 low (about 5 positive cells per 100 tumor cells, suggesting low cell kinetics) (Figure 4D–F).

Diagnosis of IM of left sartorius muscle was made.

## 3. Discussion–Literature Review

Infantile myofibroma/myofibromatosis is a rare but well recognized spindle cell neoplasm, a mesenchymal disorder caused by the fibrous proliferation in the skin, bone, muscle, and viscera [3].

Defined as a rare disease by the National Organization for Rare Disorders (NORD) and Orphanet, with an estimated incidence of 1/150,000 to 1/400,000, IM is the most common fibrous tumor in infancy and childhood [4,5].

IM was first described in 1951 as “congenital fibrosarcoma” by Williams and Schrum. Then, in 1954, Stout called it “generalized congenital fibromatosis”, suggesting a multicentric benign fibroblastic process [6]. Since this paper, other cases have been reported in the literature under different names, such as multiple hamartomas, multiple vascular leiomyomatosis of the newborn, and multiple congenital fibromatosis. In 1965, Kauffman and Stout defined two forms: one involving skin, subcutaneous tissue, or skeleton, without visceral involvement, having a good prognosis; another type, affecting soft tissue, muscles, bones, and internal organs, with poor prognosis [7]. In 1981, a study was conducted by Chung and Enzinger to review the cases reported so far and determine certain features of the disease that remain in place [8]. The authors changed the name from congenital generalized fibromatosis (CGF) to “infantile myofibromatosis” and also established the cell lines from which the lesion appeared and defined the differences in prognosis with the location and involvement, solitary or multicenter, with or without visceral compromise [8]. Finally, in 1989, Smith et al. and Daimaru et al. coined the terms “myofibromas” and “myofibromatosis”, nomenclature adopted by the World Health Organization (WHO) to describe solitary form (myofibroma) or multicenter form (myofibromatosis) [9,10].

Infantile myofibromatosis (IM) has a male predominance of 1.7/1 and low incidence. As the lesions may not be clinically visible and involute spontaneously, the condition may be underdiagnosed and underreported. The lesions can appear over a wide age range, but generally manifest in infancy, half of them being diagnosed at birth; about 90% of cases are diagnosed before the age of 2 years [3,5].

The etiology of the disease is still unknown, the majority of the cases reported being sporadic and isolated; there seems to be a familial pattern suggesting an autosomal dominant or recessive trait [11,12]. In the later years, two genes have been identified as causing the disease: PDGFRB and NOTCH3. Mutation in the receptor of the platelet derived growth factor (PDGFRB), a tyrosine kinase receptor, are mitogens for cells of mesenchymal origin. The process is regulated by NOTCH3 [12]. Cytogenetic and fluorescence in situ hybridization analyses showed a pseudodiploid karyotype with an interstitial deletion of the long arm of chromosome 6, del (26) (12q15q), which was the sole anomaly found thus far [13].

Prenatal diagnosis of an IM was first reported in 1998 by Kubota et al., describing a lesion of the triceps detected at 36 weeks of gestation [14]. As with all soft tissue lesions, IM can be diagnosed during pregnancy, the optimal time for detection being between 30 and 32 weeks; even so, the majority of the cases were diagnosed later during pregnancy (36–38 weeks of gestation). A careful prenatal US examination in the second part of the gestation is generally able to detect this type of lesion, but there are also reports of utilizing fetal MRI for a better definition of the abnormal tissue, to assess the extent and to exclude multicentric visceral forms. The prenatal imaging findings are not pathognomonic, but MRI is superior in differentiating fibrosarcoma. Generally, IM was reported in the literature as having prenatal diagnosis with visceral involvement, in which case, if the lesions were multiple and involved important viscera, pregnancy termination could be an option [14,15,16,17,18,19]. Our case was recognized as a soft tissue tumor relatively early in the pregnancy and well described as a unique tumor, with a less frequent localization of IM.

Clinically, a firm or hard nodule in the skin, subcutaneous tissue, muscle, or bone is defined as the solitary form, which is the most common (50–75% of cases), as noted by Chung and Muraoka, being the form of presentation in our patient [8,20]. The single nodules of myofibroma are usually well circumscribed, with an initial phase of rapid growth, and painless [9]. It mainly affects the regions of the head, neck, and torso. In the case presented, it had localization at the lower limb in a female patient, which is type that is much more common in males, especially in the upper part of the body.

Multiple lesions define the multicentric form, with or without visceral involvement. The multicentric form usually presents at birth and the solitary form may present later, usually in the first 2 years of life [5,21]. Wiswell reported that 37% of multicentric localization had visceral involvement [22].

Imaging is not pathognomonic but serves to assess the degree of disease (especially in multifocal forms), disease progression, and diagnosis of recurrences [15]. The ultrasound examination can reveal a hyperechoic or anechoic center with a surrounding ring, and offers information about the stiffness, encapsulation, liquefaction, calcification, and blood flow. In soft tissue tumors, CT exam will show isodense or less dense than muscle, with defining characteristics such as peripheral enhancement and calcifications; in forms with bone involvement, the image will be similar to the radiographically aspects, showing well defined osteolytic lesions with sclerotic rings. MRI reveals mass with low signal center and peripheral rim enhancement on T1-weighted [23].

The differential diagnoses for soft tissue masses includes neurofibroma, sarcoma, metastatic neuroblastoma, and other neoplasms (like congenital childhood fibrosarcoma and inflammatory myofibroblastic tumor), dermoid or epidermoid tumors. Histological examination of biopsies or resected tumors remains the gold standard method for diagnosis [24].

Microscopically, both the solitary and multicentric forms have similar characteristic appearances with a distinct zoning pattern. There are spindle cells arranged in whorled or interlacing fascicles, giving a leiomyoma-like appearance in the peripheral areas of nodular lesions. The constitutive spindle cells demonstrate staining characteristics of both myoblasts and fibroblasts, and frequently contain a large quantity of collagen within the surrounding matrix. In the central areas, a hemangiopericytoma-like pattern consisting of cells with less differentiation is usually found. A high mitotic rate with the presence of up to as many as 3 mitotic figures per 10 high-power field and infiltration of adjacent adipose tissue and skeletal muscle are not unusual, but have no adverse prognostic significance [8,22,25,26]. As in our case, biphasic tumor proliferation consisting of nodules with necrosis and central calcifications, and between the nodules, a fusiform cell-proliferate arranged in small intersecting bundles, were the main feature.

The prognosis and natural evolution depend on the type of IM. Spontaneous regression may occur in solitary cases and also may be observed in most of the multicentric cases, within 18 to 24 months. In this scenario, careful follow-up must be done, as recurrences after regressions may occur [22]. Chung et al. reported a 7% recurrence rate [4]. Massive apoptosis has been suggested as a mechanism of tumor regression [26].

The treatment should be individualized, as there are no standard protocols or guidelines because of the rarity of the disease. The interventions, procedures and therapeutics are variable according to the number, size, location, symptoms, and evolutive pattern of IM.

Solitary lesions or multiple lesions in infants, in the absence of visceral involvement, usually have a benign and self-limited course that ends with spontaneous regression. If the tumor size increases significantly in a short time or if it produces compression to surrounding structures, surgical removal may be considered. The recurrence rate after excision in the solitary form is less than 10% [22,27]. In the case presented, the rapid and constant growth of the lesion before and after birth, along with the particular characteristics (prenatal diagnosis, female, lower limb mass, and muscle localization), and the schwanoma-like appearance at MRI dictated surgical excision.

In cases of persistent nodules or recurrence and in forms with visceral involvement when surgery was not possible, chemotherapy, radiation, and steroid therapy have been tried for treatment of IM [28,29]. In addition, subcutaneous interferon alfa (3 million U/m^2^ daily) for 2 months was the treatment in a Turner’s syndrome patient with IM, which resulted in a decrease in size and apoptosis on histological examination [30].

A connection between the p.R561C mutation in gene encoding platelet-derived growth factor receptor beta (PDGFR-beta) and the development of infantile myofibromatosis, was confirmed by recent studies, raising the idea that PDGFR-beta phosphorylation in tumor cells may be reduced by the receptor tyrosine kinase inhibitor sunitinib, a very promising agent that affects the proliferation of tumor cells with a p.R561C mutation in PDGFR-beta [31].

The case reported was particular because it associates some rare features: prenatally diagnosed solitary lesion in a female patient, with localization in the lower part of the body. We have systematized the cases identified in the literature by prenatal diagnosis, their features, treatment, and outcomes (Table A1).

## 4. Conclusions

IM is a differential diagnosis of soft tissue masses in early infancy and should be considered despite its low frequency and even if the typical characteristics are not met, like in the case presented. For diagnosis, the biopsy is mandatory, as treatment and prognosis depend on lesion characteristics such as clinical form (solitary or multicentric), location, and visceral involvement.

## Appendix A

**Table A1:** Cases with prenatal diagnsosis, their characteristics, management and outcome.

PRef	GA (Weeks)	Fetal Gender (M/F)	Location & Size (cm)	Prenatal Investigation (Type & Findings)	Management	Outcome Follow Up
Nishioka, 1999 [32]	37	M	Chest wall (5.0 × 5.5 × 2.5)	US: tumor recognized on the chest wall.	Resection and skin graft at 7 days of life.	No recurrence at 11 months.
Kubota, 1999 [14]	36	F	Left upper arm (8 × 7 × 5)	US: solid and spherical, slightly inhomogeneous, moderately echogenic mass, well demarcated with no evidence of bone invasion.	Resection of tumor at 2 months of age.	No evidence of tumor or functional disorder 3 years after surgery.
Meizner, 2000 [15]	30	F	Paraspinal (4.2 × 7.5 × 3.5)	US (30 w): solid mass on the left side of the spine extending from T7 to L5 of the vertebral column; no Doppler blood flow. MRI (31 w): well-defined mass, no direct connection between the mass and spinal cord elements.	Pregnanacy termination (32 w) via fetocide injection.	On autopsy, a large lump on the left side of the spine, with no connection to the spinal canal. Visceral involvement was also noted in the liver and retroperitoneum.
Wataganara, 2007 [33]	35	F	Anterior fetal neck, extending onto the anterior chest wall (10.8 × 9.2)	US (35 w): homogeneous solid mass. MRI: in midsagittal plane, a huge solid mass on the anterior portion of the neck and chest was observed, with low signal intensity mass. Nasopharynx, oropharynx, and trachea intact and filled with amniotic fluid position.	Spontaneously ruptured membranes at 35 weeks. EXIT procedure Resection at 11 days of age.	At 7 months of age, no signs of recurrence.
Muraoka, 2008 [20]	32	F	Spleen (5.7 × 3.9)	US (32 w): abdominal tumor, 5.7 × 3.9 cm; (34 w): grown to 6.0 × 5.5 cm. MRI (36 w): left upper abdominal tumor.	Splenectomy on day 20 of life.	No recurrence at 3 years of age.
Arabin, 2009 [18]	13	F	Superficial head	Monoamniotic twins. 13 w: echodense tumor on the side of one twin’s head. 18 w: vascularization of the tumor. 30 w: intestinal dilatation (same twin). MRI: excluded connections to the ventricles or brain.	Superficial head tumor resected and laparotomy for abdominal mass.	Affected twin died from sepsis 12 days after birth; Unaffected twin normal at follow up.
Yeniel, 2013 [34]	32	F	Left lung (4.6 × 2.7)	US: solid mass detected in the parenchyma of the left lung; Color Doppler: avascular.	Thoracotomy and complete mass resection on day 2 of life because of respiratory distress syndrome.	No recurrence or symptoms 1 year after surgery.
Zhang, 2014 [17]	38	M	Right side paraspinal, a pseudoulcerate plaque (2.4 × 2.2 × 1.2)	US: mass on the fetal right back (T3-8), hypoechoic with sporadic hyperecho. Color Doppler flow: intermittent blood flow inside and around the mass. MRI (next day after US): normal result, with no mass.	Resection of the mass 3 months after birth.	No recurrence at 2 years after surgery.
Coleman, 2016 [35]	29	F	Posterior medial aspect of the left thigh of her fetus (3.1 × 3 × 3.8 at 29 w; 5.6 × 4.8 × 5.3 at 33 w)	US (29 w): First visualization. US (33 w): Mass increased in size, hypoechoic compared to the adjacent subcutaneous fat, but isoechoic compared to muscle. The underlying bone was intact. MRI (same day with the last echo) defined the mass as relatively well-circumscribed, superficial location that replaced the medial subcutaneous tissues of the thigh.	Surgical excision on hospital day 5 without complication, though it was found to involve the investing fascia of the gracilis and sartorius muscles. No direct involvement of the major neurovascular structures.	No functional or strength deficits in the lower extremity at the age of 9 months.
Vemavarapu, 2017 [36]	32	F	Left arm (9 × 5)	US: well circumscribed mass, arising from the left arm, just beside the humerus, but the osseous structure normal; Color Doppler: moderate blood flow in the solid part of the mass. MRI: improvised the detection and evaluation.	Resection of tumor.	Normal upper function and no recurrence 1 year after surgery.
Pekar-Zlotin, 2018 [37]	34	-	Multiple tumors	US: multiorgan involvement, with masses predominantly in the lower extremities, heart, abdominal cavity, and neck. Normal Doppler studies in the major fetal arterial and venous circulations, as well as normal amniotic fluid volume and unremarkable appearance of the placenta.	Inoperable tumors.	The infant died of cardiac failure 30 days after birth.
Rekawek, 2019 [16]	36	M	Lower thoracic spine (3.5 × 2.3)	US: avascular, heterogeneous mass of soft tissues, without cord involvement. MRI: thick-walled partly cystic lesion in the right paraspinal muscles of the lower thorax.	Biopsy on day 7 of life.	MRI at one year of age: multiple soft masses decreased in size compared to prior imaging, and three additional lesions: crus of the left diaphragam, left posterior chest wall, and left shoulder.
Evens, 2021 [38]	33	-	Visceral organ involvement Lung masses	US: dilated bowel loop. MRI: suggested the dilated loop of bowel was colon, given the internal high T1 signal compatible with meconium. Additionally, multiple, solid-appearing lung masses were noted, which had not been previously visualized on ultrasound. After thorough review of the MRI images of the entire fetus, no primary lesion was found. No masses were visualized in the extremities.	The baby had not passed meconium after 1 day of life, therefor an exploratory laparotomy: nodules on the bowel serosa, resulting in a bowel obstruction. Owing the visceral organ involvement, chemotherapy was considered; however, it was ultimately decided to observe the patient and treat only if there was radiologic progression and/or clinical deterioration.	
